# Temporal meta-optimiser based sensitivity analysis (TMSA) for agent-based models and applications in children’s services

**DOI:** 10.1038/s41598-024-59743-8

**Published:** 2024-04-20

**Authors:** Luke White, Shadi Basurra, Abdulrahman A. Alsewari, Faisal Saeed, Sudhamshu Mohan Addanki

**Affiliations:** 1 College of Computing and Digital Technology, Birmingham City University, Birmingham, B4 7XG UK; 2 Antser LTD, 4 Vicarage Court, Birmingham, B15 3ES UK

**Keywords:** Mathematics and computing, Computational science, Computer science, Software

## Abstract

With current and predicted economic pressures within English Children’s Services in the UK, there is a growing discourse around the development of methods of analysis using existing data to make more effective interventions and policy decisions. Agent-Based modelling shows promise in aiding in this, with limitations that require novel methods to overcome. This can include challenges in managing model complexity, transparency, and validation; which may deter analysts from implementing such Agent-Based simulations. Children’s Services specifically can gain from the expansion of modelling techniques available to them. Sensitivity analysis is a common step when analysing models that currently has methods with limitations regarding Agent-Based Models. This paper outlines an improved method of conducting Sensitivity Analysis to enable better utilisation of Agent-Based models (ABMs) within Children’s Services. By using machine learning based regression in conjunction with the Nomadic Peoples Optimiser (NPO) a method of conducting sensitivity analysis tailored for ABMs is achieved. This paper demonstrates the effectiveness of the approach by drawing comparisons with common existing methods of sensitivity analysis, followed by a demonstration of an improved ABM design in the target use case.

## Introduction

Within Local Authority Children’s Services in the UK, the current and predicted economic outlook^1^ has increased pressure on resources and the strategic planning capabilities available. Thus, there has been renewed discussion on the improvement of the decision-making processes within local authorities (LAs) and calls for significant policy changes^2^. The impact of the COVID-19 pandemic has also been keenly felt by local authorities, with substantial changes to practice being made within Children’s Services across the UK^3^.

Existing literature within Children’s Social Care frequently proposes policy changes as drivers for improvement in services and outcomes for Children in Care^2,4–7^. Examination and evaluation of these proposals, in the context of existing practices would provide LAs with further informed decision-making to lead future policy and practice development. Despite existing data quality issues present within these contexts^8^, there is an appetite within LAs for improved understanding of data and the effects of policy decisions made clear by projects such as the Local authority interactive tool (LAIT) produced by the Department of Education^9^. This understanding of data is crucial for LAs to be able to measure service performance quantitatively. Furthermore, providing understanding between LAs would further benefit each authority, as demand is often shared between authorities.

Agent Based Modelling^10^ is a prominent and popular technique within social sciences and used to conduct analysis of social systems, such as the Linked Lives model for examining parent-child relationships^11^. This technique shows promise in relation to Children’s Services due to a focus on individual level interactions which could be related to practice and service delivery in LAs by social workers and others^12^. Utilising Agent Based Models (ABMs) would expand the tools used by data analysts within LAs as per recommendations made in the reports such as the Missing Numbers in Children’s Services^8^ and others^13^, namely the increase in data and data science literacy. Such potential has been explored recently in a previous work by using an ABM designed to emulate a Local Authority Children’s Services, which was calibrated with a Genetic Algorithm against real-world data^14^.

Simulations in general also provide some additional advantages that align with challenges identified within the Missing Data in Children’s Services report^8^, including the establishment of a common framework of analysis. With a shared understanding of analysis in place, various roles within Children’s Services could provide extensive domain knowledge to guide and inform simulation design and validation. Such collaboration may lead to more effective models of LA operations that could be utilised by the sector^15^. Furthermore, simulations provide a route for analysts to combine other available data to produce complex insights that may leverage areas present within LA remit such as, demographics, socioeconomic factors, social determinants, budgets, and workforce^8,15^.

There are some additional benefits that ABMs specifically provide, including focusing on granular agent interactions and ‘micro’ level attributes that could correlate with aspects of social worker practice and child behaviours^12^. Additionally, the implementation of ABMs within LA analysis teams could be completed effectively using existing libraries for Python, such as Mesa^16,17^. A highlighted challenge within LAs from the Missing Data in Children’s Services report^8^ and others^13^, was the improvement of data science practices and literacy. The creation of such new tools would aid in this challenge.

An integral part of creating ABMs is the validation of the model design against real-world data. Part of this can include the use of Sensitivity Analysis to interpret model behaviour^18^. Sensitivity Analysis methods look to analyse a given model to understand how changes in model input can result in changes in model output, and importantly how significant those changes in output can be. The results of such analysis provide a measure for each input parameter of the given model, with a higher value indicating that the input parameter has a stronger impact on the output of the model. Conversely, a lower value can indicate that the input parameter has little impact on the model output, which may then demonstrate that the processes related to that input parameter are not having the intended impact on model behaviour. This is useful for developing an understanding of model behaviour and robustness, of which for ABMs, is important for model validation and for model refinement^19^. Refinement in particular would then involve the application of fixes to the issues identified in the analysis leading to a new iteration of the model design to meet expected behaviours. Whilst these methods do offer approaches to better understand ABMs, there are limitations with existing methods of Sensitivity Analysis. This includes the inability to model non-linear dynamics in model behaviour, which are frequently seen in ABMs and allows them to model complex systems.

Frequently seen methods of Sensitivity Analysis include One Factor at a Time (OFAT), Regression Analysis, and Sobol’ Method^18,20–22^. The latter examples: Regression Analysis and Sobol’ Method are considered Global Sensitivity Analysis methods, where the model is examined across the full parameter space which would be of particular use for creating a holistic understanding of model behaviour.

Examining these two methods more closely, Regression Analysis is often conducted with linear models to enable interpretation of the ABM interactions through coefficients^20^. This therefore presents a limitation in the existing approaches used in regression-based global sensitivity analysis as ABMs often have non-linear interactions between model inputs and outputs^19^, making the analyses less reliable. Variance based methods such as Sobol’ Method do not demonstrate the same limitations. However, Sobol’ does have shortcomings related to sampling and computational complexity^18,19^. In order to conduct robust sensitivity analysis it is therefore observed that multiple methods should be employed to provide the best analyses possible. It is therefore desirable to enable the use of non-linear models within Regression-based Global Sensitivity Analysis, as to accompany other methods such as Sobol’ for understanding ABMs.

Observing the current literature on sensitivity analysis methods, regression-based approaches are conducted in a similar process to typical machine learning tasks. Within machine learning there is often additional processes conducted on top of the original modelling workflow to further improve performance, this can be referred to as Meta-optimisation. Meta-optimisation^23^ can be seen as the technique or set of techniques employed in some common machine learning related tasks, such as hyper-parameter tuning or feature selection; often to improve the performance and training time of the subsequent model. Furthermore, the term meta-optimisation refers specifically to the use of an optimiser to optimise the parameters of another optimiser. The wider term for such methods and tasks is Automated Machine Learning^24^.

Meta-optimisation can be conducted using various algorithms, which may include genetic algorithms^25^, swarm algorithms^26^, and simulated annealing^27^. The choice of which algorithm to use for a given meta-optimisation task may be dependent on the specifics of the task, however the aforementioned methods have been seen to be used to improve machine learning model performance^28–30^.

Thus, a possibility arises whereby it is desired to understand the significance of an ABM’s parameters (Sensitivity Analysis) and this need can be seen akin to Feature Selection, a meta-optimisation task, where model parameters act like features in a dataset. Feature selection^31^ is often performed to reduce the size of training data^32^, which can increase model performance by removing features that may be considered either redundant or irrelevant. Using this same process, it is plausible that a metric of parameter significance can be derived from feature selection and used as an approach to sensitivity analysis.

Such a method of sensitivity analysis would resolve the issues identified previously by enabling the use of non-linear models within a regression analysis approach. This can be done as the internal machine learning workflow would use a non-linear model and a meta-optimiser would run atop this to identify the most significant parameters of the target ABM in a feature selection type process. This method would accompany using Sobol’ to provide a more robust analysis of a given ABM and provide insight necessary for the improvement of the target ABM design. This method in conjunction with ABMs generally would subsequently be beneficial for analysts in Children’s services as models can then be created, analysed, and refined more effectively by analysts to provide more insights and extract additional value from existing public data.

Thus, presented in this paper, will be a sensitivity analysis method designed to accommodate the non-linear nature of ABMs. By leveraging a feature selection approach, a novel sensitivity analysis metric can be created that allows for non-linear models to be used to conduct the analysis, aiding in better understanding a given ABMs behaviour and to more robust analysis when combined with other methods such as Sobol’.

## Results

**Figure 1:**
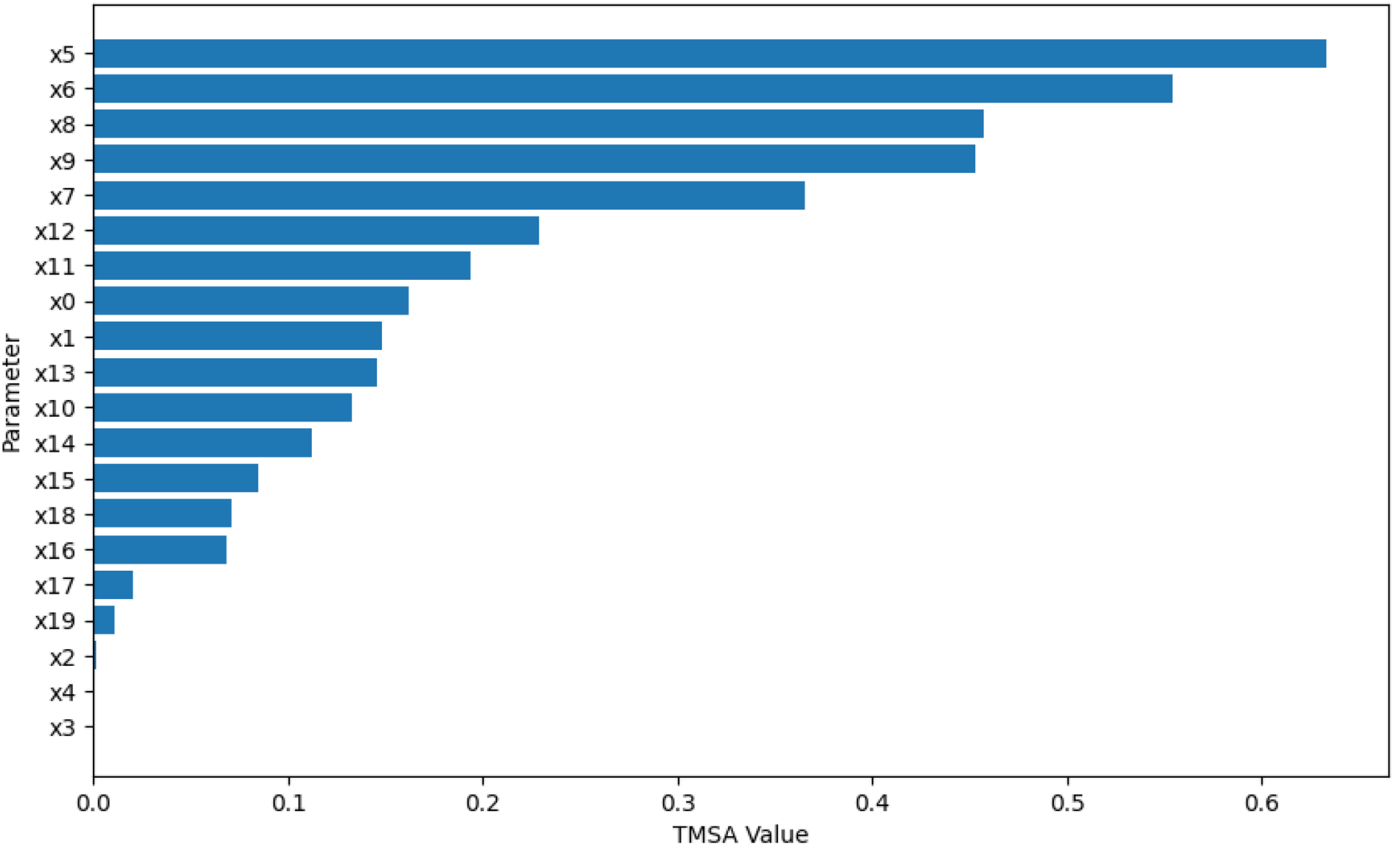
TMSA values averaged over all six model outputs and 364 time-steps.

**Figure 2:**
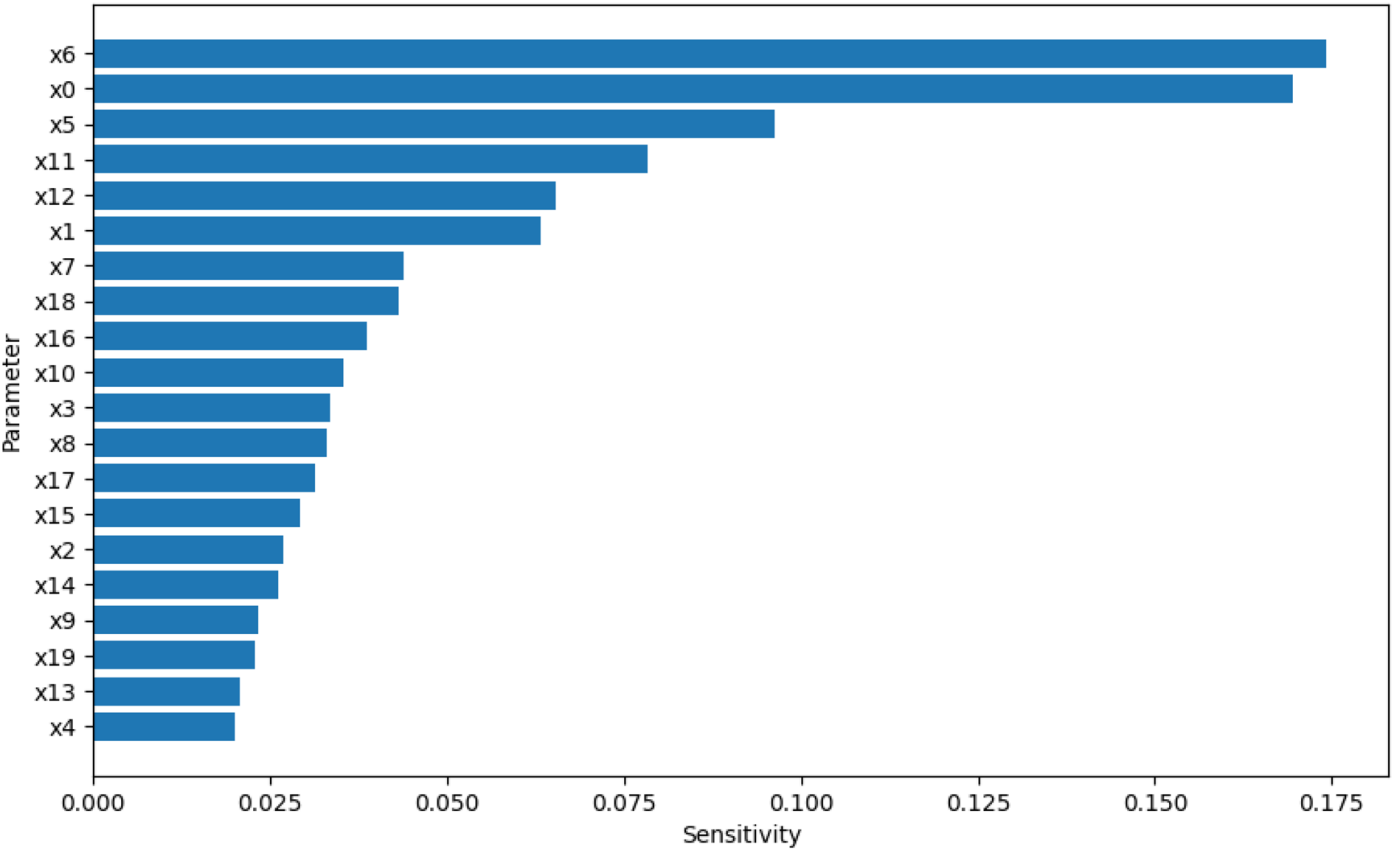
SOBOL First-order sensitivities averaged across all six model outputs and 364 time-steps.

**Figure 3:**
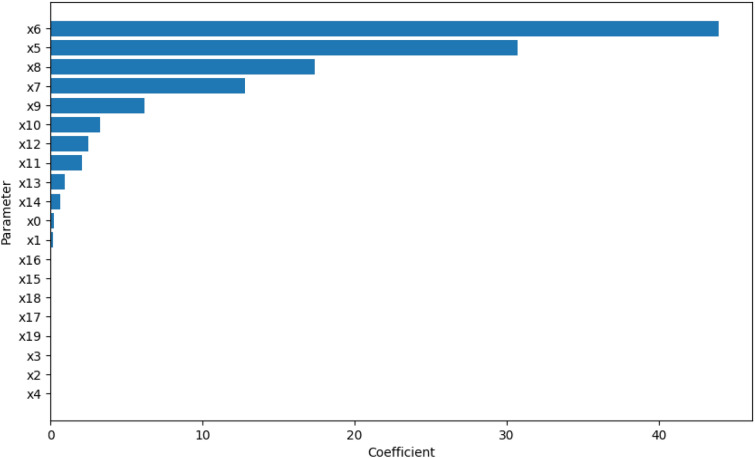
Linear Regression coefficients averaged over all six model outputs and 364 time-steps.

Using the design and methodology outline in this paper, results were collected from the proposed method called Temporal Meta-optimiser based Sensitivity Analysis (TMSA), and the selected existing methods of Linear Regression and Sobol’ method. These three methods were used to conduct sensitivity analyses against a target ABM. Figures 1, 2 and 3 present the outputs from these methods totalled over the full target simulation period, consisting of 364 steps to align with a single year of real-world data. Table 1 further displays these results as it presents a full comparison of the methods when measured over the full simulation period. As can be seen, TMSA ranks the target ABMs parameters comparably to both Linear Regression and SOBOL.

**Table 1:** Comparison of values and ranking of parameters between LR, SOBOL, and TMSA.

Parameter	LR		SOBOL		TMSA	
	Value	Rank	Value	Rank	Value	Rank
x0	0.23	11	0.17	2	0.16	8
x1	0.14	12	0.063	6	0.15	9
x2	0.0054	19	0.027	15	0.0014	18
x3	0.0063	18	0.034	11	0.00046	20
x4	0.0025	20	0.020	20	0.00092	19
x5	30.73	2	0.096	3	0.63	1
x6	43.95	1	0.17	1	0.55	2
x7	12.78	4	0.044	7	0.37	5
x8	17.40	3	0.033	12	0.46	3
x9	6.17	5	0.023	17	0.45	4
x10	3.25	6	0.035	10	0.13	11
x11	2.07	8	0.078	4	0.19	7
x12	2.46	7	0.065	5	0.23	6
x13	0.96	9	0.021	19	0.15	10
x14	0.64	10	0.026	16	0.11	12
x15	0.047	14	0.029	14	0.085	13
x16	0.050	13	0.039	9	0.069	15
x17	0.015	16	0.031	13	0.020	16
x18	0.017	15	0.043	8	0.071	14
x19	0.0075	17	0.023	18	0.011	17

**Figure 4:**
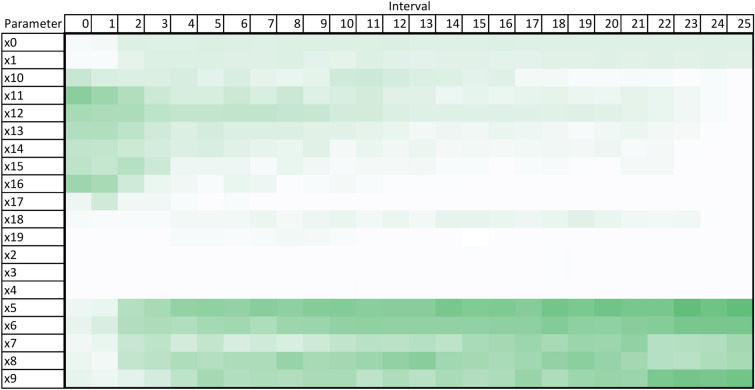
TMSA values over the full simulation period.

**Figure 5:**
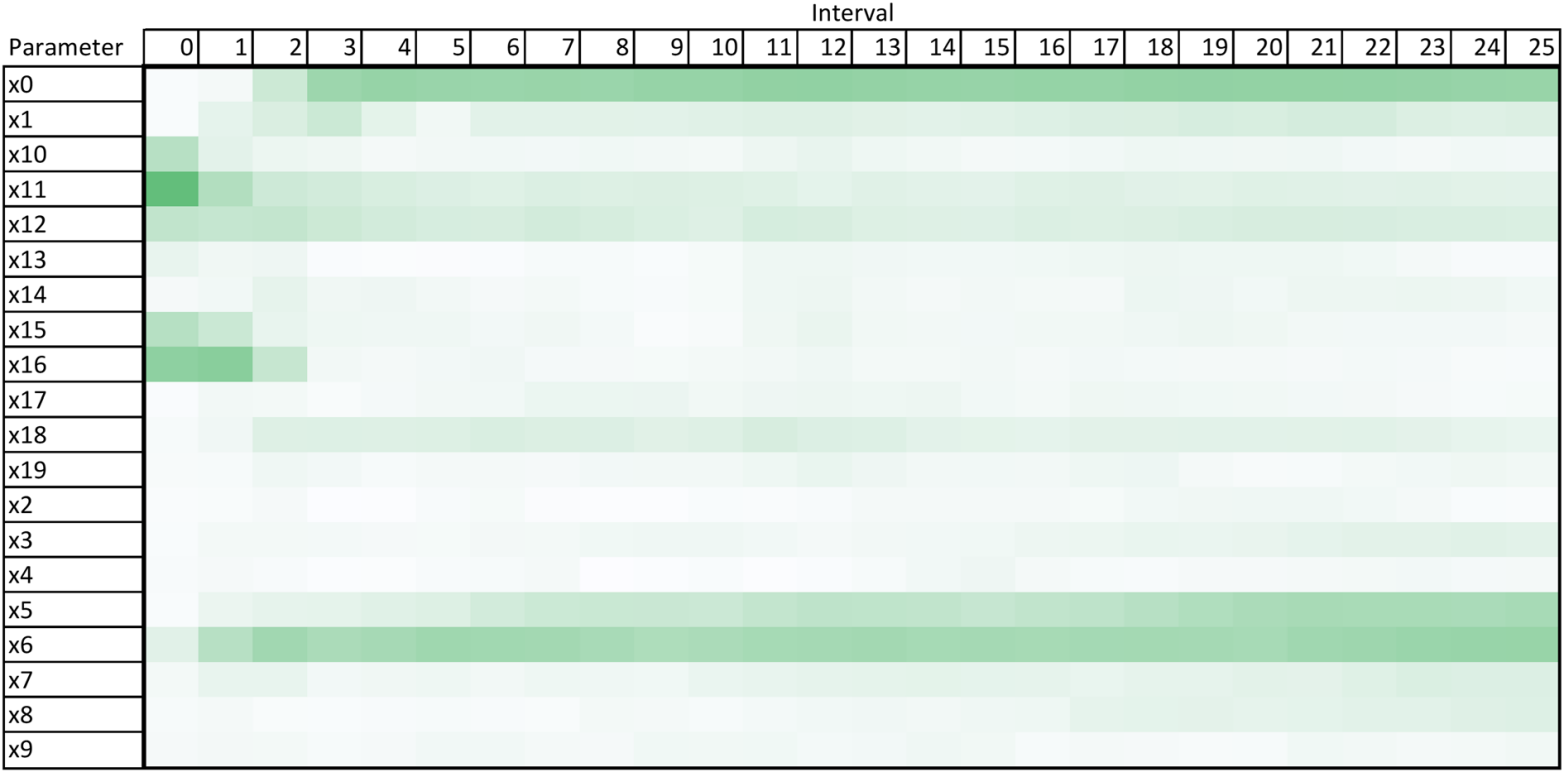
SOBOL first order sensitivities over the full simulation period.

**Figure 6:**
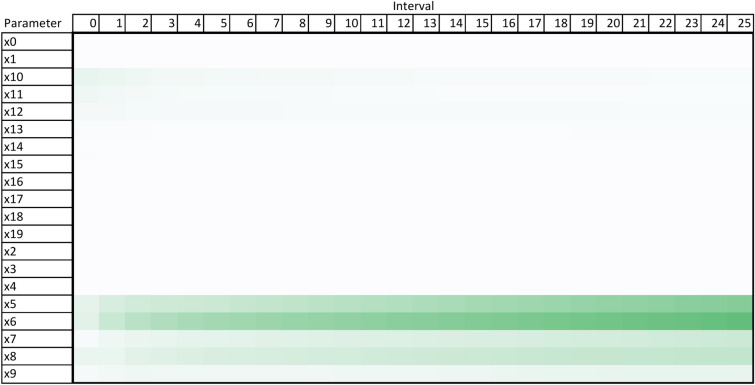
Linear Regression coefficients over the full simulation period.

Following on from this, Figs. 4, 5 and 6 present more granular data over the full 364 time-steps, divided into 26 intervals (14 time-steps each) to provide a heat-map of the TMSA/SOBOL values over the simulation period.

As can be seen in Figs. 4 and 5, TMSA indicates that more of the parameters are having significant impact on the model output in comparison to SOBOL, which highlights a small number of parameters as significant with the remaining parameters having a lower significance. Linear Regression appears to show a small group of parameters with high significance but many others offer little to no significance. All methods signal a similar group of parameters, demonstrating consistency between TMSA and the existing methods.

TMSA shows two distinct groupings of parameter significance in Fig. 4, namely a group with high significance during the first 3 intervals and another group with high significance for the remainder of the simulation. These groups correspond directly to groups of parameter types within the design of the ABM, namely the pre-allocation parameters which have the most impact at the start of the simulation; the other grouping being the conversion rate parameters, which have the most impact for the rest of the simulation. SOBOL identifies a parameter from each group within the design as significant, however it does not then mark other parameters within that group with the same significance. Linear Regression highlights the second group of parameters similar to SOBOL, but shows a lower significance on the remaining parameters.

However, there is some observed differences between TMSA and SOBOL of note. There is another group of parameters in the ABM, namely x0 through x4, which govern the length of a given type of case within the simulation. These parameters are given some uniform significance by TMSA, however SOBOL gives a high significance to one parameter in this group (x0) and the others a lower significance. This may be explained by the stochastic elements of the meta-optimisation algorithm used in TMSA (NPO) whereby these parameters are being selected nearly uniformly due to the random initial values used to initialise the NPO algorithm for each time-step analysis. This can also be seen in the other parameter group mentioned previously, x5 through x9. Thus, it can be seen that SOBOL, in the case of the ABM analysed here, appears to highlight a single parameter from each grouping due to their similar significance on the output but mainly due to the deterministic nature of SOBOLs calculation. This may provide an advantage to the use of TMSA over SOBOL as this effect results in easier identification of parameters with shared effects on the output of the target ABM, whether intended by design or not.

### Validation against use case

Using these figures, it was clear that some ABM parameters relating to average case length (x0 through x4) were not having any significant impact on the model output, namely: x2, x3, x4 which relate to the case lengths of Children in Need (CIN), Child Protection Plan (CPP), and Children in Care (CIC) cases. Furthermore, all parameters relating to pre-allocation ranges (x15 through x19) saw little significance other than in the first time-step interval. This also applied to a lesser extent to the other pre-allocation parameters relating to the limits in the number of each type of case that can be pre-allocated (x10 through x14). The most significant parameters were conversion rates (x5 through x9) and the length of referrals and assessments (x0 and x1). The less significant processes and parameters mentioned previously were reworked and or removed completely from the ABM design. This led to a smaller set of parameters (15), fewer agent processes, and a reduction in computation time to run the model itself. With the reworked model completed, the TMSA process was conducted again and yielded the following outputs.

**Figure 7:**
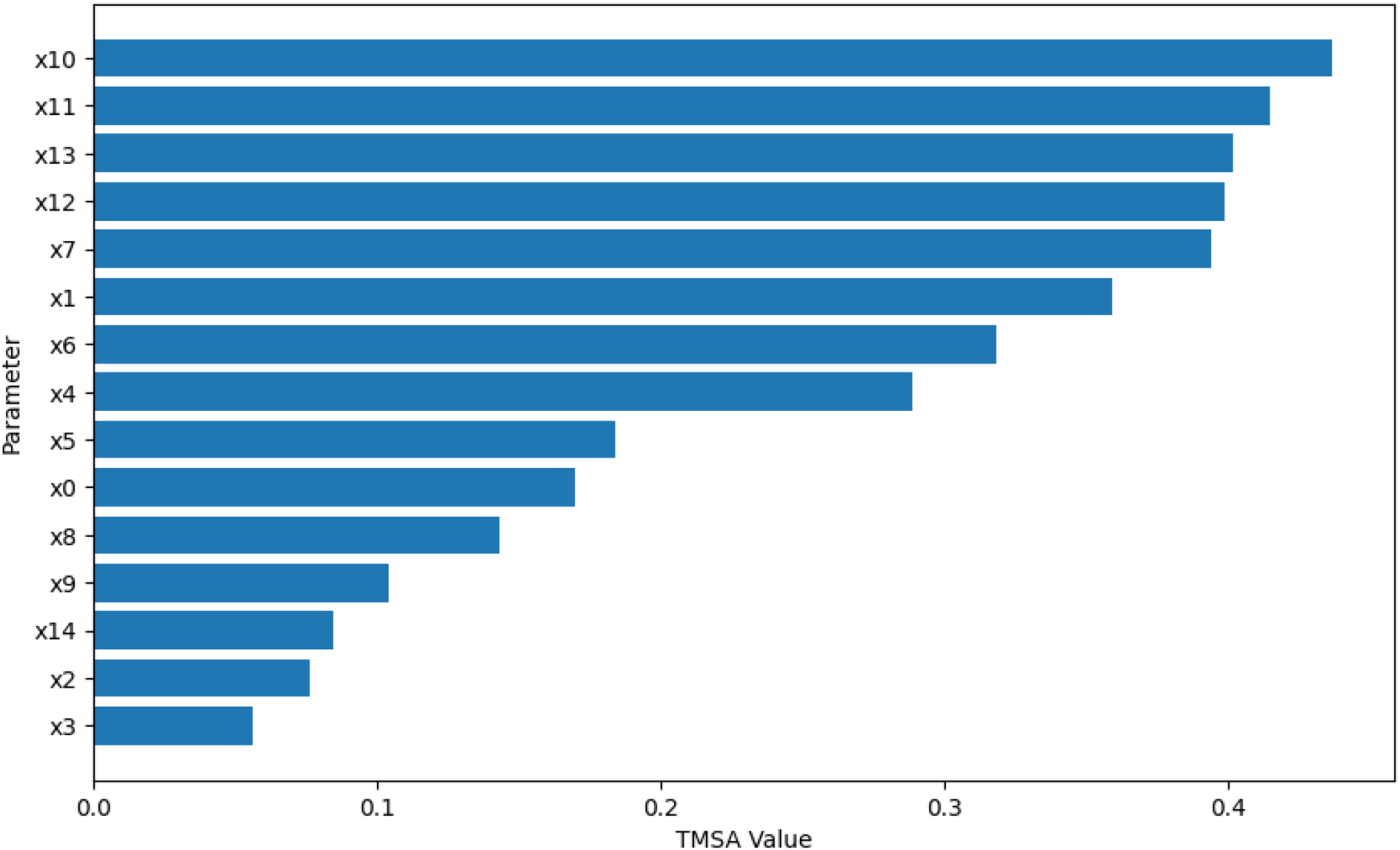
TMSA values averaged over all six model outputs from the redesigned ABM.

**Figure 8:**
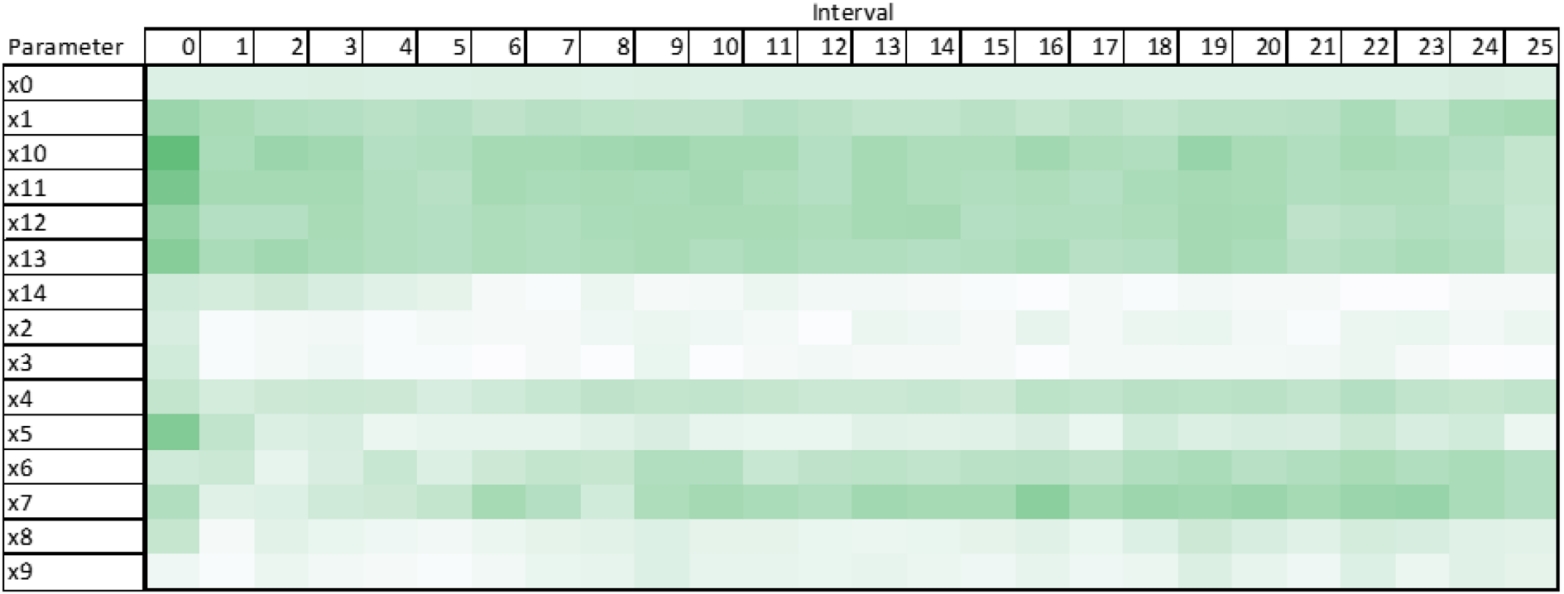
TMSA values over the full simulation period from the redesigned ABM.

It is clear from Figs. 7 and 8, that the reworked ABM has parameters that have greater significance for more of the simulation period. Within these outputs, none of the reworked parameters has a low measure of significance to the model output.

The ABM was originally designed to use an accompanying process to calibrate it’s parameter values to a given LAs real-world data. With this reworked ABM this calibration process was run and compared to the previous model, with the results presented in Table 2.

**Table 2:** Results of comparison between the original ABM Average full-time worker caseload (AFWC) mean squared error (MSE) errors (AFWC previous) from the calibration process, and the reworked ABM design (AFWC rework).

Local authority	AFWC previous	AFWC rework	Change
Warrington	0.23	0.60	+ 0.37
Darlington	3.93	1.38	– 2.55
York	0.05	0.002	– 0.048
Rutland	0.003	0.35	+ 0.347
Herefordshire	0.27	0.008	– 0.262
Luton	0.0005	0.11	+ 0.1095
Hammersmith & Fulham	0.004	0.14	+ 0.136
Merton	4.0	1.05	– 2.95
Bracknell Forest	0.03	0.026	– 0.004
Bath & NE Somerset	0.03	0.12	+ 0.09
Average	0.852	0.3786	– 0.4734

**Table 3:** Computation time in seconds (S) of local authority simulations using the original ABM design and the re-designed ABM, conducted over 1000 sample runs each.

Local authority	Original ABM (S)	Re-designed ABM (S)	Improvement
Warrington	0.430	0.335	22.10%
Darlington	0.234	0.208	11.11%
York	0.299	0.249	16.72%
Rutland	0.159	0.125	21.38%
Herefordshire	0.373	0.268	28.15%
Luton	0.459	0.329	28.32%
Hammersmith & Fulham	0.358	0.290	18.99%
Merton	0.370	0.292	21.10%
Bracknell Forest	0.271	0.210	22.51%
Bath & NE Somerset	0.308	0.247	19.81%
Average	0.326	0.255	21.02%

The redesigned ABM was also able to improve upon computation time: the original ABM required 0.159 seconds to execute a full simulation period, whilst the redesigned ABM required 0.125 seconds to execute. This represents a 21.38% improvement and was conducted via an average of 1000 sampled ABM runs based on Rutland local authority. The full set of sample authorities were also examined for computation time, the results of which may be found in Table 3. From the compute times, it is clear that the redesigned ABM provided a significant improvement over the original with and average reduction in compute time of 21.02%.

## Discussion

With the results demonstrated, the proposed method appears to present a comparable analysis to the existing methods of Sobol and Linear Regression. Namely, the most highlighted parameters of the ABM align with each of the analysis methods, therefore aiding in validating the analysis produced by the TMSA method.

When examining the most significant parameters provided by TMSA compared to the other methods, TMSA appears to highlight contextually related parameters with a comparable amount of significance. This is most evident in the aforementioned parameters x5 through x9, as these are all used within the Social Worker case management process within the ABM. This propensity for TMSA to highlight complete processes may be of importance for identifying key processes within a target ABM that can be further examined to improve the overall ABM design. This was used to great effect in the results presented.

A key area identified in the literature was the use of linear models within regression-based sensitivity analysis, thus presenting potential difficulties when examining ABMs due to their non-linear design. The results presented demonstrate that the proposed TMSA method provides valid and useful analysis for a given ABM example. While further investigation can be conducted to further verify this method, it is clear that TMSA provides a potential novel sensitivity analysis method that enables the use of non-linear regression models for more effective regression-based sensitivity analysis. There are some limitations to the current implementation of the TMSA method used in this experiment, namely that the time to conduct the analysis was significantly slower than the other methods used. This could be remedied by a more optimal implementation of the optimisation algorithm used: the Nomadic Peoples Optimiser. Despite this, along with further investigation into using other optimisation algorithms for potential improvements, the TMSA method shows promise for the intended target contexts. The overall performance of the TMSA method shows that this approach may be of significance for furthering ABM analysis techniques, especially when in conjunction with existing methods such as Sobol.

Through the results presented, the proposed TMSA method has potential value as an additional sensitivity analysis approach. With the advantages outlined previously, TMSA could complement analyses made on ABMs specifically, which could be largely beneficial for use cases where ABMs may be the preferred modelling approach. Namely for children’s services in the UK, the improvement of methods for analysing ABM behaviour would allow for greater confidence in and adoption of ABMs for use cases such as demand modelling, policy evaluation, and forecasting.

Further work into this area could yield further techniques that would be of value to contexts such as children’s services, whereby using analysis methods such as TMSA, processes and behaviours within the ABM could be specifically identified as being influential on model output. This could be further extended to propose changes that suggest behaviour alterations to align with real-world data more, using a process such as calibration. This could be beneficial, especially to children’s services, where existing assumptions could be examined and possible unexpected behaviours could be identified and investigated.

Overall, with further pressure on children’s services in the UK and demand for more effective data analytics capabilities, TMSA may provide a potential approach that can accompany ABMs to create additional value for children’s services from existing models and data. Additionally, the work presented can act as a tool in future investigations for both ABMs and the further development of novel methods of ABM analysis and validation.

## Methods

In order to examine and evaluate the potential for the proposed approach, an experimental methodology was used where an example ABM will be examined with the proposed method and compared to other common approaches, as identified previously in the literature.

The selected ABM was developed to simulate a LA children’s services, which was used in previous work with a calibration process to optimise parameter values to allow the ABM to produce outputs in line with publicly available data^14^. The ABM is designed with two agents: an administrative Children’s Social Care Services (CSCS) agent, and a Social Worker agent. The CSCS Agent’s designed processes could be summarised as: the delegation of new work (Referrals) to Social Workers, and the handling of LA statistics. The Social Worker Agent’s processes can also be summarised as: the handling of new and existing cases assigned to themselves, and the pre-allocation of existing work based on the current caseload. The design of the ABM was based on the statutory guidance provided by the Department of Education regarding Children’s Services^33^.

**Figure 9:**
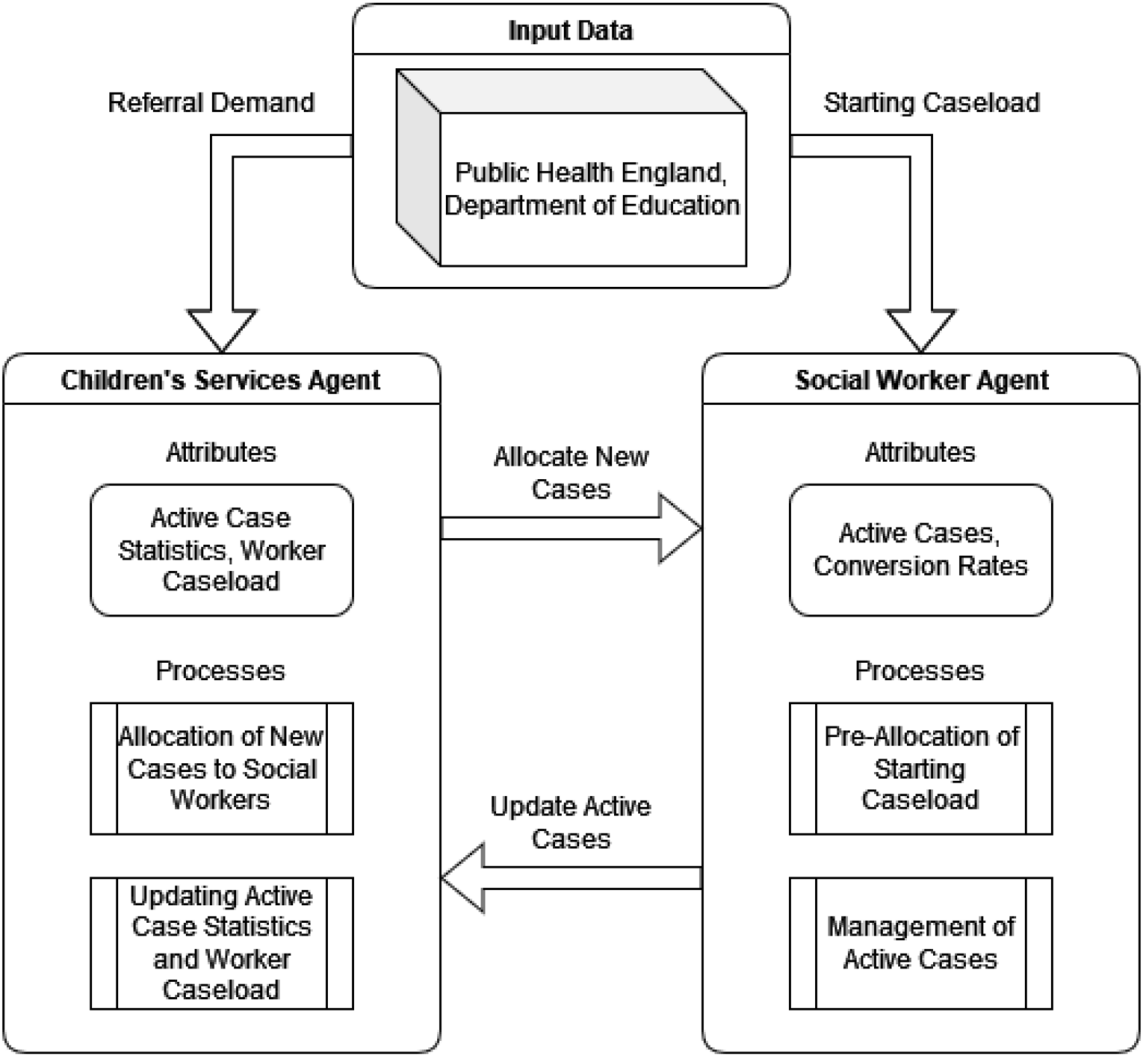
High-level view of simple local authority ABM.

Figure 9 summarises the aforementioned design and expected behaviour from each agent type. Furthermore, the parameters of the original ABM that were grouped into differing application areas: Environmental, Case Lengths, Conversion Rates, Starting Case Limits, and Starting Case Age. This can be seen in Table 4.

**Table 4:** Configurable parameters of the original ABM.

Parameter	Symbol
Average referral length	*x*0
Average assessment length	*x*1
Average CIN length	*x*2
Average CPP length	*x*3
Average CIC length	*x*4
Referral no further action rate	*x*5
Assessment no need rate	*x*6
CIN conversion rate	*x*7
CPP conversion rate	*x*8
CIC conversion rate	*x*9
Pre-allocation referral limit	*x*10
Pre-allocation assessment limit	*x*11
Pre-allocation CIN limit	*x*12
Pre-allocation CPP limit	*x*13
Pre-allocation CIC limit	*x*14
Pre-allocation referral range	*x*15
Pre-allocation assessment range	*x*16
Pre-allocation CIN range	*x*17
Pre-allocation CPP range	*x*18
Pre-allocation CIC range	*x*19

The agents processes in the original ABM were designed to be simple whilst following the aforementioned guidance. There were four key processes in the ABM, two for each agent type. For the CSCS agent these consisted of the allocation of new referral cases to workers, and the calculation of worker statistics with the management of workers should they need to be removed or added to the simulation.

Referral allocation was simulated by using the amount of daily referrals, supplied by the real-world data, and randomly adding new referrals to the list of cases that each worker agent has.

The addition and removal of Worker agents that is managed by the CSCS agent is also determined from the real-world data. The difference in social workers from the previous simulation step to the current step is used to determine if the CSCS agent creates or removes social worker agents from the ABM. If a worker agent is removed then the cases assigned to that worker are then redistributed randomly to other workers. If however, a new worker agent is added, then cases will be randomly taken from existing workers to provide the new worker with a list of cases. The calculation for the difference in number of worker agents, and the full management algorithm can be seen in Eq. (1) and Algorithm 1 respectfully. *SW* represents the number of worker agents in the simulation.

1 $$\begin{aligned} diff_{SW} = SW_{Current} - SW_{Previous} \end{aligned}$$

**Algorithm 1:**
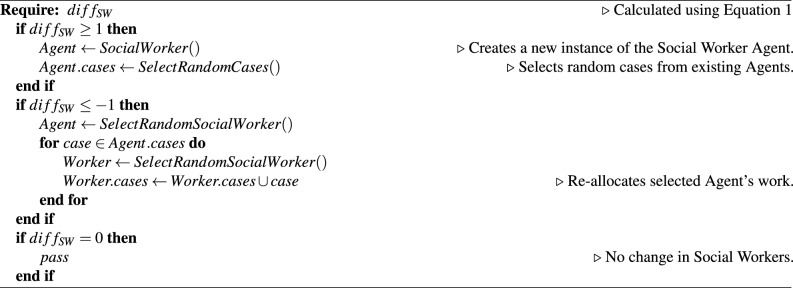
Social worker agent management algorithm.

For the Social Worker agent the designed processes consisted of the pre-allocation of cases at the beginning of the simulation to represent existing cases, and the management of cases for each time-step.

In the case of the pre-allocation process, the number and age of the cases is determined using the limit and range parameter respectfully for each case type. The age of an existing case cannot exceed the age described in the associated length parameter. Algorithm 2 demonstrates how this age is calculated for the referral case type.

**Algorithm 2:**
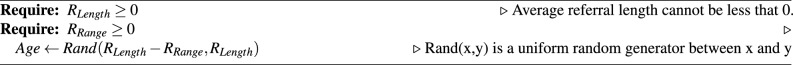
Age of pre-allocated referrals algorithm.

To simulate the workflow of Social Workers within the ABM, each case is processed and actions are decided using the aforementioned length and conversion rate parameters. If the age of a case exceeds the respective length parameter, then the case may advance to the next case type, subject to the probability set by the respective rate parameter, otherwise it is removed from the case list. Once a case has been processed and remains on the list, the age of the case is increased by 1 day. Algorithm 3 outlines this process with the referrals case type.

**Algorithm 3:**
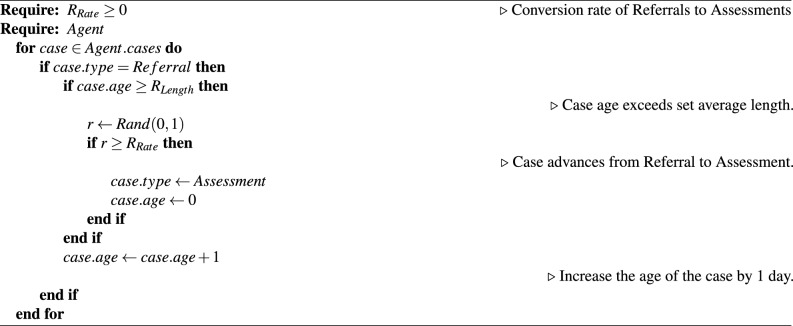
Referrals case management algorithm.

The original ABM produces 6 output values per time-step, these include: average worker caseload, active referrals, active assessments, active children in Need (CIN) cases, active child protection plans (CPP), active children in care cases (CIC). In the case of the proposed method, the average worker caseload will be analysed, as this is the main target output of the ABM.

This ABM was designed with the intention of being flexible in order to model for the variety of LAs within the UK. However, limitations were identified during the previous evaluation when using the accompanying calibration process^14^. Namely, the ABM failed to find as optimal solutions for 2 of the 10 sample authorities: Darlington and Merton.

The approach used to examine the effectiveness of the proposed TMSA method will consist of multiple analyses of the existing example ABM, using the previously identified common sensitivity analysis methods. These will then be compared against TMSA and the ABM will then be re-designed based on the conclusions of the TMSA analysis to validate the findings.

### ABM dataset generation

To conduct the proposed analysis method, a dataset is required. The dataset is generated from a sample number of full simulation runs, with each simulation consisting of 364 time-steps to represent a year of time. Each simulation in the sample was created by using a random selection of parameter values within an upper and lower bound for the value of each parameter. The complete sample contained 1000 simulations and was used as the dataset for evaluation of the proposed method.

The design of the dataset can be seen as per Table 5. For each time-step of a given sample run, we store the output values from the ABM, in the case of the Local Authority Model there were 6 outputs included in the dataset. From these we selected a target output that the evaluation of the proposed method would be conducted against, which was the Average Full-time worker Caseload (AFWC). The final dataset was stored in a Comma Separated Values (CSV) file and the algorithm used is described in Algorithm 4.

**Table 5:** Example of the dataset generated from the sample runs of the ABM, with *t* denoting the time-step of the data.

x0	...	x19	t	AFWC	Refs	Assess	CIN	CPPs	CICs
35	...	720	0	17.0	89	56	218	0	0
35	...	720	1	19.1	94	57	230	2	0
35	...	720	2	20.1	89	59	233	2	0
35	...	720	3	20.1	86	61	237	2	0
35	...	720	4	20.2	83	65	241	2	0

**Algorithm 4:**
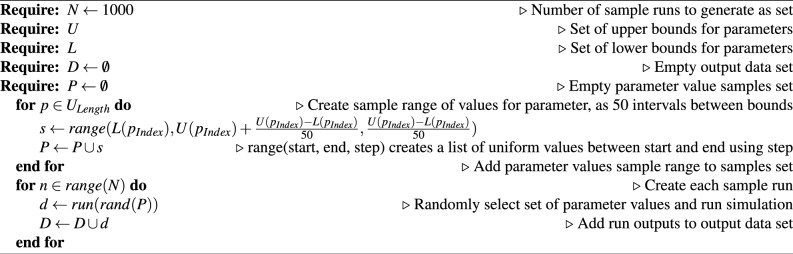
Data generation using target ABM.

### Meta-optimiser based sensitivity analysis

The proposed method seeks to create a novel approach to Sensitivity Analysis by viewing the analysis akin to a Feature Selection problem. In Feature Selection it must be determined the most optimal set of features for typically a machine learning model to use in its training process, whereby selecting the most optimal features can improve the trained models performance significantly. We therefore draw parallels to the questions Sensitivity Analysis looks to answer, namely which parameters have the most significance to a given models output; and to the questions Feature Selection looks to answer, namely identifying the optimal features for improving trained machine learning model performance.

Thus, our proposed method seeks to generate a Sensitivity Analysis metric from using an implementation of Feature Selection. The method uses a target ABM and its parameters and identifies the optimal selection of those parameters to train a machine learning model. The score produced by the machine learning model is used to identify how optimal a candidate set of parameters is. This process is conducted over each time-step of a target simulation period, and by taking each optimal set of parameters found per time-step, it can be calculated how frequently each parameter appeared to create a final Sensitivity Analysis metric based on how often the parameter appeared. This would result in a metric where when a parameter appeared more in the sets of optimal parameters, it will receive a higher value, implying a higher significance to the target ABMs output.

To facilitate the optimal selection of parameters for the machine learning model, we use a meta-optimiser. The meta-optimiser will take an objective function, which in this case will be to minimise the error produced from the machine learning model, and identify the set of parameters that achieves the lowest loss.

To evaluate the proposed method, we select a meta-optimisation algorithm and a machine learning model to be trained. The machine learning model selected for this evaluation is a K-Nearest Neighbour Model (KNN), chosen for its non-linearity and fast computation speed, which runs through a 5-fold cross-validation (CV) of a train/test split of the generated dataset mentioned previously, to ensure that the performance of the trained model is measured accurately.

The algorithm chosen to act as a meta-optimiser for this evaluation is the Nomadic Peoples Optimiser (NPO)^34^. This was selected due to its comparatively quick convergence time and its minimal need for configuration. These traits are desired by the target context, Children’s Services, due to inexperience with such techniques^35^. Other optimisation algorithms may be used depending on contextual needs.

**Figure 10:**
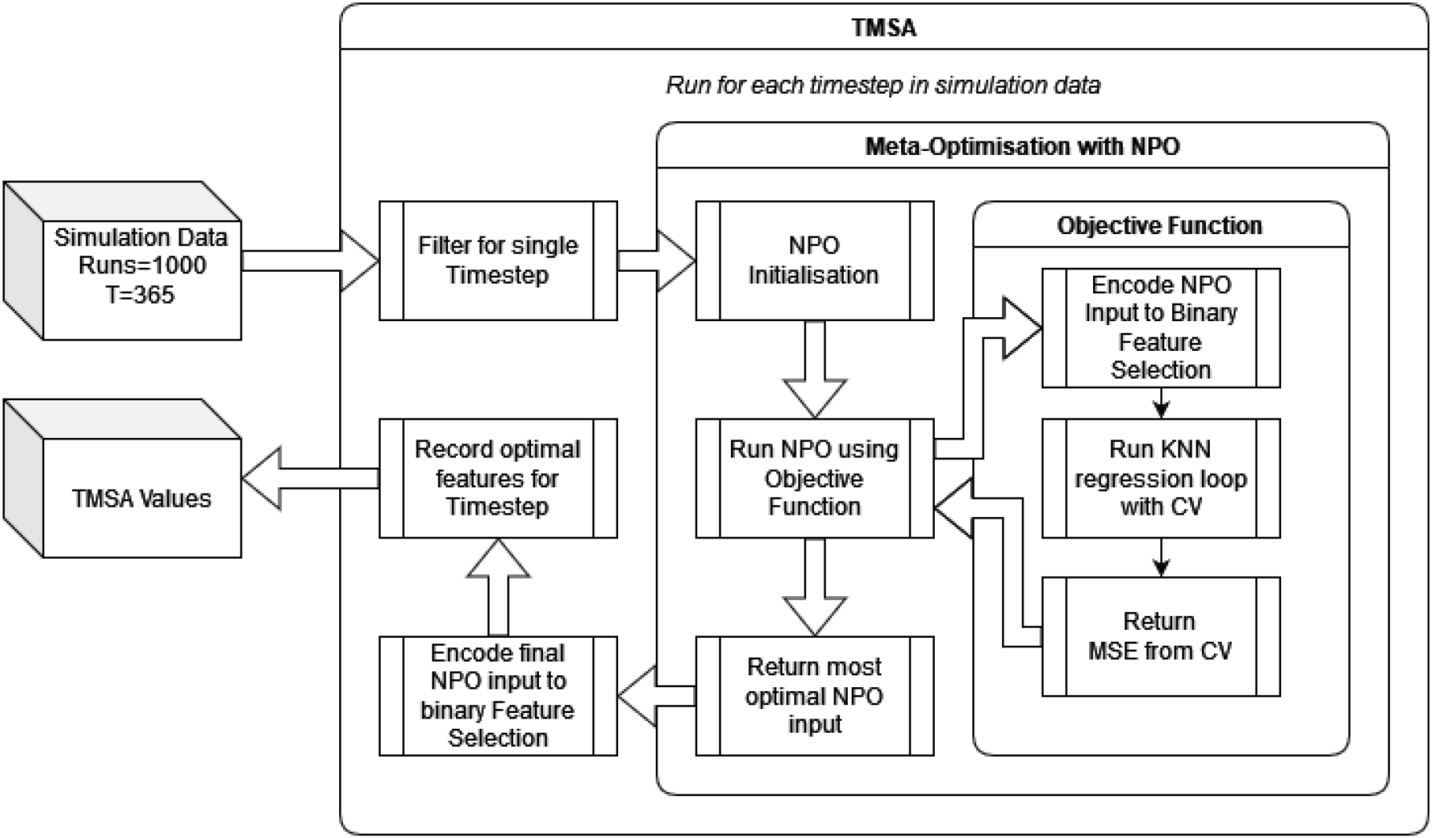
Proposed meta-optimisation based approach.

With these choices in mind, a high level overview of the process can be seen in Fig. 10, with the algorithm of the proposed method in Algorithm 6 with the objective function defined in Algorithm 5.

**Algorithm 5:**
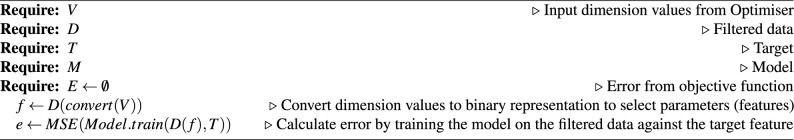
Objective function algorithm.

**Algorithm 6:**
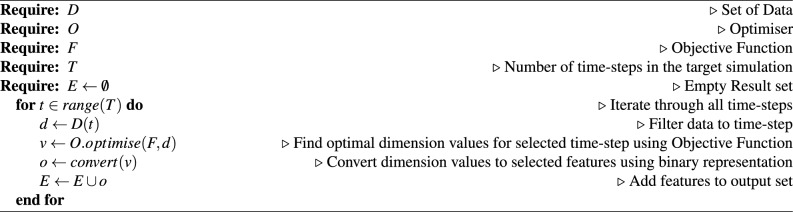
Temporal meta-optimiser based sensitivity analysis algorithm.

For the NPO algorithm to be able to select candidate sets of parameters for the KNN model, an additional step is needed to convert the floating point (decimal) values that NPO generates which will be referred to as Dimension Values, to a discrete set of binary values (either 0 or 1) to then be able to determine the parameters to include in the KNN model training. This was done using the algorithm outlined in Algorithm 7.

This process uses whole integer division to enable a binary representation to be created, this is then used to flag each feature to be used within the internal machine learning model training. The calculation for this division can be seen in Eq. (2), which returns a set of selected features, based on an input set of dimension values and the set of features available. The dimension values represent the selected combination of features to be used in training the internal machine learning model, such that the combinations from a large search space can be selected within the limitations of the software implementation. This reduces the possible combinations by partitioning the parameter search space into a desired number of dimensions. This was necessary as a means to convert the decimal value that the NPO algorithm uses for a given dimension into a binary value that further translates to a feature selection. Having only one dimension for searching was also sub-optimal; in the implementation, the decimal number is directly mapped to its binary equivalent. Thus, to search for all 20 parameters in the model within a single dimension value, the boundaries for that value would range from 0 to $2^{20}$. Should there be 4 dimensions used instead, then each dimension would correspond to a boundary range of 0 to $2^5$. This provides a smaller search space for the NPO algorithm to optimise within and then allows TMSA to accommodate models with significantly more parameters.

An example of the output this algorithm would be as per Table 6.

**Table 6:** Examples of the conversion of decimal dimension values to a binary representation to enable Feature Selection.

Dimension values	Number of features	Binary output
(11.2)	4	$1011(11) = 1011$
(3.0, 2.0)	4	$11(3), 10(2) = 1110$
(480.4, 780.7)	20	$0111100000(480), 1100001101(781) = 01111000001100001101$
(10.3, 16.7, 8.1, 25.8)	20	$01010(10), 10001(17), 01000(8), 11010(26) = 01010100010100011010$

**Algorithm 7:**
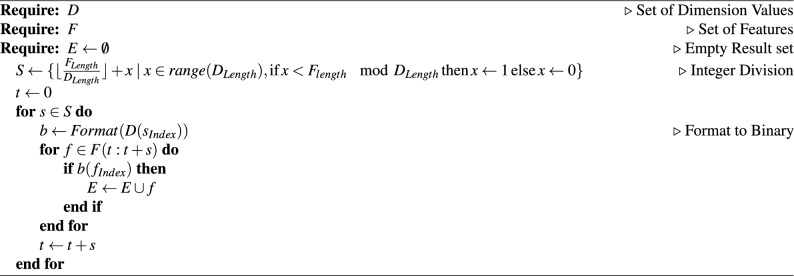
Binary encoding for feature selection algorithm.

Once the optimal feature selections have been found for each time step in the simulation period, the TMSA values can be calculated. In the case of calculating the values over the entire simulation period, the frequency of a given parameter appearing in optimal selections is divided by the total simulation period, as per Eq. (3).

2 $$\begin{aligned} Divide(D, F)&= \left\{ \left\lfloor \frac{F_{Length}}{D_{Length}} \right\rfloor + x \mid x \in range(D_{Length}),\ \text {if}\, x < F_{length}\,\mod \, D_{Length}\,\text {then}\, x = 1\, \text {else}\, x = 0\right\} \end{aligned}$$

3 $$\begin{aligned} TMSA(P)&= \frac{Frequency(P)}{Period} \end{aligned}$$

For a more granular visualisation of the TMSA values over the simulation period, the gathered selection data can be divided into intervals and the same process applied, whereby the TMSA value would be calculated over the interval rather than the entire simulation period. These interval TMSA values can then be visualised as a heat map to demonstrate the changes in TMSA values over the simulation, as per Fig. 4.

### Comparisons

In order to compare TMSA with other methods such as SOBOL, the same analyses are conducted as described previously. In the case of SOBOL, sensitivity indices are calculated for each time step and model output of the simulation period. First order sensitivities will then be examined using the same visualisations for the entire simulation period and for each interval period to provide granularity.

### Calibration use case

To validate the proposed TMSA process, the calibration use case that the original ABM was developed for was used. Using the TMSA process, the original ABM is analysed to identify limitations with the design and then modified to address the found limitations. The aforementioned calibration process will then be conducted and the performance of the new design will be compared to the original ABM design.

Calibration refers to the optimal adjustment of a given ABMs parameters to best match a desired output from that model, by using an automated approach that can take advantage of optimisation algorithms such as Genetic Algorithms (GA). In the case of the ABM used in this comparison, it was desired that the ABM produce outputs to closely match real-world data that was publicly available, such as the Average Full-time Worker Caseload (AFWC) seen previously. This process is needed due the variety in which LAs behave differently, thus it is impractical to manually determine the best values for the model parameters to represent a given authority.

The calibration process consists of a GA based multi-objective optimisation of model parameter values to real-world data, through 10 sample LAs. The performance of the fit of parameter values is conducted using Mean Squared Error calculations on the model outputs compared to the real-world data. The GA used in the case of the calibration process, was NSGA-III^36^, as this was found to be the most performant algorithm through iterative experimentation. It can also be seen in the literature that NSGA-III is likely more suitable for multi-objective optimisation tasks^37^. The objective function used in the process measures 10 different objectives, which measure the variance of the model outputs against the real-world data and ensure any unreasonable outputs are penalised, such as having no remaining social care cases within the simulation.

## Data Availability

The datasets generated during and/or analysed during the current study are available from the corresponding author on reasonable request.

## Code availability

The code created during the current study are available from the corresponding author on reasonable request.
